# Electrophysiological evaluation of an anticancer drug gemcitabine on cardiotoxicity revealing down-regulation and modification of the activation gating properties in the human rapid delayed rectifier potassium channel

**DOI:** 10.1371/journal.pone.0280656

**Published:** 2023-02-02

**Authors:** Mengyan Wei, Pu Wang, Xiufang Zhu, Masaki Morishima, Yangong Liu, Mingqi Zheng, Gang Liu, Hiroki Osanai, Kenshi Yoshimura, Shinichiro Kume, Tatsuki Kurokawa, Katsushige Ono

**Affiliations:** 1 Department of Cardiology, The First Hospital of Hebei Medical University, Shijiazhuang, Hebei Province, People’s Republic of China; 2 Department of Pathophysiology, Oita University School of Medicine, Yufu, Oita, Japan; 3 Department of Food Science and Nutrition, Faculty of Agriculture, Kindai University, Nara, Japan; Xuzhou Medical University, CHINA

## Abstract

Gemcitabine is an antineoplastic drug commonly used in the treatment of several types of cancers including pancreatic cancer and non–small cell lung cancer. Although gemcitabine-induced cardiotoxicity is widely recognized, the exact mechanism of cardiac dysfunction causing arrhythmias remains unclear. The objective of this study was to electrophysiologically evaluate the proarrhythmic cardiotoxicity of gemcitabine focusing on the human rapid delayed rectifier potassium channel, hERG channel. In heterologous hERG expressing HEK293 cells (hERG-HEK cells), hERG channel current (*I*_*hERG*_) was reduced by gemcitabine when applied for 24 h but not immediately after the application. Gemcitabine modified the activation gating properties of the hERG channel toward the hyperpolarization direction, while inactivation, deactivation or reactivation gating properties were unaffected by gemcitabine. When gemcitabine was applied to hERG-HEK cells in combined with tunicamycin, an inhibitor of N-acetylglucosamine phosphotransferase, gemcitabine was unable to reduce *I*_*hERG*_ or shift the activation properties toward the hyperpolarization direction. While a mannosidase I inhibitor kifunensine alone reduced *I*_*hERG*_ and the reduction was even larger in combined with gemcitabine, kifunensine was without effect on *I*_*hERG*_ when hERG-HEK cells were pretreated with gemcitabine for 24 h. In addition, gemcitabine down-regulated fluorescence intensity for hERG potassium channel protein in rat neonatal cardiomyocyte, although hERG mRNA was unchanged. Our results suggest the possible mechanism of arrhythmias caused by gemcitabine revealing a down-regulation of *I*_*hERG*_ through the post-translational glycosylation disruption possibly at the early phase of hERG channel glycosylation in the endoplasmic reticulum that alters the electrical excitability of cells.

## Introduction

Gemcitabine is an antineoplastic drug, which is commonly used particularly to treat several types of solid organ malignancies such as pancreatic cancer, non-small cell lung cancer, and breast cancer [1]. One of the drug profiles that differentiate antineoplastic pharmacological therapies from other pharmacological therapies is the frequency and severity of adverse effects at clinical application. The most common adverse effects of gemcitabine are symptoms in gastrointestinal, hepatic and hematopoietic disorders including nausea, vomiting, increased alanine aminotransferase (ALT) and aspartate aminotransferase (AST), increased alkaline phosphatase, anemia, and thrombocytopenia [2]. Furthermore, gemcitabine is also known to cause cardiovascular adverse reactions including myocardial ischemia, pericardial diseases, heart failure, and arrhythmias [2]. Among them, supraventricular tachyarrhythmias, especially atrial fibrillation often occur in cancer patients with gemcitabine therapy [3, 4]. There is increasing recognition that the association of gemcitabine with atrial fibrillation is strong and can occur even after the first dose, suggesting that gemcitabine could interact with arrhythmogenic substrates in the heart [5]. Drug interactions with cardiac ion channels are the underlying mechanisms for the occurrence of arrhythmias. Although rare, association of gemcitabine with QT interval prolongations in electrocardiogram (ECG) was also reported in several studies [6–9]. QT interval prolongation is actually not considered to be a toxic substrate, however, this electrical derangement has been observed in human subjects just prior to the onset of cardiac arrhythmias. Co-occurrence of QT interval prolongation and arrhythmias has led to a postulated link between QT and arrhythmogenic ion channel dysregulation. One of the primary causes of drug-induced QT interval prolongation is thought to be blockage of the hERG potassium channel in cardiac myocytes [9]. Of note, multiple recent studies have concluded that prolonged QT interval has an increased risk of atrial fibrillation [10–14].

In the present study, we aimed to electrophysiologically evaluate the action of gemcitabine as a potential modulator of cellular excitability focusing on the hERG channel. We examined effects of gemcitabine on the human embryonic kidney 293 (HEK293) cells stably expressing the hERG channel (hERG-HEK cells) but not on cardiomyocytes because of the following reasons. 1) Freshly isolated cardiomyocytes from adult animals are not suitable for the patch clamp study after the incubation for 24–48 h, 2) Rapid delayed rectifier potassium channel current (I_Kr_) are very low in current density in neonatal rat or mouse cardiomyocytes [15], and 3) Our preliminary study revealed that immortalized cells such as H9c2 demonstrate very different electrophysiological features in comparison with cardiomyocytes (data not shown). Since these disparities usually indicate complexed multiple actions of drugs on ion channel transcription, translation, and/or trafficking to the plasma membrane, this study’s purpose was to increase understanding of the arrhythmogenic actions of gemcitabine focusing on the hERG channel at the post-transcription stage or later by use of hERG-HEK cells, exploring possible mechanism for atrial fibrillation and QT interval prolongation as a major cardiotoxicity of this drug.

## Materials and methods

The experimental protocol was approved as “a non-animal study” in advance by the Ethics Review Committee for Animal Experimentation of Oita University School of Medicine.

### Cell preparation

All experimental protocols were approved in advance by the Ethics Review Committee for Animal Experimentation of Oita University School of Medicine (No. C004003, No. G004006), and were carried out according to the guidelines for animal research of the Physiological Society of Japan to minimize the number of animals used, as well as their suffering.

The human embryonic kidney 293 (HEK293) cells stably expressing the hERG channel (hERG-HEK cells) are generous gifts from Professor Imaizumi, Nagoya City University, Japan. hERG-HEK cells were cultured in Dulbecco’s modified Eagle’s medium (DMEM, Hyclone, Logan, UT, USA) with 15% fetal bovine serum (FBS, Gibco) at 37°C and exposed to an atmosphere of 5% CO_2_. The culture medium was also supplemented with 400 μg·mL^-1^ gentamycin (G418, Calbiochem, USA). hERG-HEK cells were treated by gemcitabine for 5 min (short-term effect) in the recording chamber of patch clamp or for 24–48 h (long-term effect) in the cell culture medium for detection of the drug actions on *I*_*hERG*_ and the hERG channel gating properties. Neonatal rat ventricular myocytes were prepared from 1- to 2-day-old Wistar rats as described previously [15]. The cardiomyocytes were maintained at 37°C under 5% CO_2_ in Dulbecco’s modified Eagle’s medium (DMEM) supplemented with 10% fetal bovine serum for 24 h, then the medium was changed to medium containing gemcitabine (0.01 μM to 50 μM) or vehicle for 24–48 h.

### Electrophysiological recording

**Fig 1:**
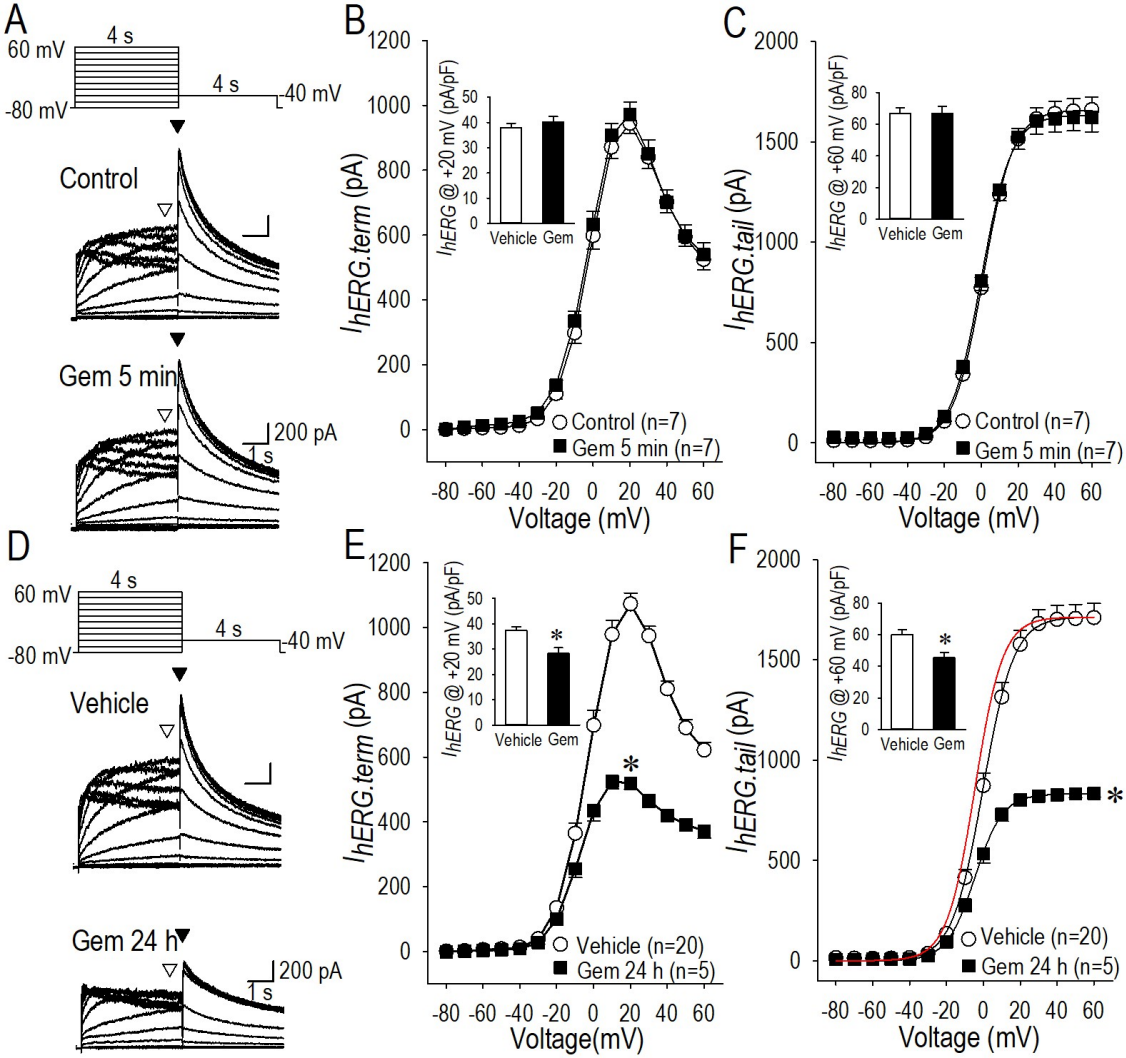
Acute- and long-term effect of gemcitabine on *I*_*hERG*_ or rapidly activating delayed rectifier potassium current (*I*_*Kr*_). (A) Representative *I*_*hERG*_ family traces in the control and during the acute (5 min) application of gemcitabine (Gem) (50 μM) are shown. Pulse protocol is shown above. (B) The relationship between terminal *I*_*hERG*_ (*I*_*hERG*.*term*_) measured at the end of each test pulse indicated by hollow arrowheads in panel A (▽) and the test pulse potential (V) constructed by using group data are shown as *I*_*hERG*.*term*_-*V* relationship in control (〇) and during the acute application of 50 μM gemcitabine in 5 min (■). The maximum current amplitude of *I*_*hERG*.*term*_ at +20 mV was945 ± 31 pA in control (〇), and was 972 ± 38 pA with 50 μM gemcitabine (■) in 5 min. (C) The relationship between tail *I*_*hERG*_ (*I*_*hERG*.*tail*_) measured at the beginning of the third pulse at -40 mV indicated by filled arrowheads in panel A (▼) and test pulse potential (V) constructed by using group data are shown as *I*_*hERG*.*tail*_-*V* relationship in control (〇) and during the acute application of 50 μM gemcitabine in 5 min (■). (D) Representative *I*_*hERG*_ family traces of the control and long-term (24 h) application of gemcitabine (50 μM) are shown. Pulse protocol is shown above. (E) The *I*_*hERG*.*term*_-*V* relationship constructed by using group data in control and long-term application of 50 μM gemcitabine for 24 h. The maximum current amplitude of *I*_*hERG*.*terml*_ was significantly reduced by 50% from 1072 ± 34 pA (vehicle) to 540 ± 25 pA (gemcitabine), where the potential for the maximum current was shifted to the hyperpolarized potentials from +20 mV (vehicle) to +10 mV (gemcitabine). (F) The *I*_*hERG*.*tail*_-*V* relationship constructed by using group data in control and long-term application of 50 μM gemcitabine for 24 h. The maximum current amplitude of *I*_*hERG*.*tail*_ at 60 mV was significantly reduced by 51% from 1709 ± 73 pA (vehicle) to 846 ± 17 pA (gemcitabine). Red line represents Boltzman-fitting curve of gemcitabine data normalized to the *I*_*hERG*.*tail*_ in vehicle at +60 mV. Insets in B, C, E anf F show *I*_*hERG*_ density at the potentials indicated by each panel graph ordinate. Numbers of cell are shown in parentheses. Values represent the mean ± SE. Unpaired Student’s *t* test was performed.

We used a whole-cell patch clamp technique for the measurement of *I*_*hERG*_ and action potentials according to the previous studies [16–18]. Briefly, *I*_*hERG*_ was recorded by whole-cell patch clamp using an EPC-9 amplifier controlled by Pulse ver.8 software (HEKA Eletronik, Lambrecht, Germany). Patch pipettes were pulled from 75-mm plain capillary tubes (Drummond Scientific Co., Broomall, PA, USA) by Model P-97 (Sutter Instrument Co., Novato, CA, USA), and were heat-polished subsequently to achieve the pipette resistance at 2–4 MΩ when filled with the pipette solution shown below. Series resistance was compensated by at least 80% and was continually monitored throughout the experiment. All the measurements were done at room temperature (20–23°C). For the current recording, the chamber was filled with bath solution contained (mM) NaCl 135, KCl 5, MgCl_2_ 1, CaCl_2_ 2, HEPES 10, glucose 10 (pH of 7.4 by adjusted with NaOH). The patch electrodes were filled with pipette solution consisting of (mM) KCl 135, MgCl_2_ 1, EGTA 5 and HEPES 10 (pH 7.2 by KOH). For the action potential recording, the bath solution (Tyrode’s solution) contained (mM) NaCl 140, MgCl_2_ 1, KCl 5.4, HEPES 10, glucose 10 and CaCl_2_ 1.8 (pH of 7.4 adjusted with NaOH), and the pipette solution contained (mML) KCl 140, MgCl_2_ 2, creatine phosphate 5, HEPES 10, EGTA 0.05 and Mg-ATP 5 (pH of 7.2 adjusted with KOH). For analysis of the conductance-voltage relationship or *I*_*hERG*.*tail*_-*V* relationship, tail current of *I*_*hERG*_ was evoked through a 4-s voltage step to -40 mV, following a 4-s depolarization step with a 10 mV stepwise increase from -80 to 60 mV, which was initiated after a holding potential of -80 mV (Fig 1). The peak of the outward tail current of *I*_*hERG*_ were plotted against the voltage of test pulses, and the data were fitted to the Boltzmann function, as: f(V) = 1/(1 + e[(V_1/2_−V)/k]) + C, where the V_1/2_ value is the membrane potential when the relationship reaches half level, and the k value is the slope factor. C is a constant component. For analysis of the activation gating properties, we used an envelope of tails procedure, which takes advantage of the fact that although at potentials positive to approximately +30 mV a small and brief transient component of inactivation overlaps with activation [19], recovery from inactivation is rapid [20] and deactivation is relatively slow [21]. The activation kinetics were evaluated by plotting the activating inward tail current envelope as a function of test pulse duration by use of a single exponential equation fits, which provided the time constant (τ_act_) for activation at each individual potential, based on the simple Hodgkin and Huxley model [22] (Scheme 1). Namely, hERG-HEK cells were evoked through a depolarization step, from -10 mV to +30 mV, for an incremental duration for 20ms-4,000 ms followed by a holding potential of -100 mV. At each depolarizing potential, τ_act_ was then plotted against the depolarizing voltage, yielding τ_act_-voltage relationship (Fig 3), which were further best fitted to a three-parameter single exponential function: T(x) = T_0_ + A x (exp(-x/τ)). For this equations T(x) is the time at voltage x, A is the amplitudes of the exponential, and τ is the voltage constant. For analysis of the inactivation gating properties, the inactivating kinetics were evaluated by the time constant of the tail current; hERG-HEK cells were evoked through a 2.5-s depolarizing steps (test potentials) from -60 mV to +60 mV to inactivate, following a 5-ms repolarization at -120 mV from the holding potential of +60 mV (Scheme 1). At each test potential, onset of each inactivation current or the decay of tail current during each voltage step was fitted by use of a single exponential curve, which provided the time constant for inactivation (τ_inact_) at the individual potentials, then τ_inact_ was plotted against the voltage, yielding τ_inact_-voltage relationship (Fig 4). For analysis of the deactivation gating properties, the deactivating kinetics were evaluated by fitting the deactivating inward tail current at each repolarizing voltage step (-180 mV to -110 mV) from the holding potential of +60 mV to a single exponential curve (Scheme 1), providing the time constant for deactivation (τ_deact_) at the individual potentials, then τ_deact_ was plotted against the voltage, yielding τ_deact_-voltage relationship (Fig 5). For analysis of the reactivation gating properties namely recovery from the inactivation gating properties, the reactivating kinetics were evaluated by the amplitude of peak tail current corresponding to the hyperpolarizing pulse from -200 mV to +60 mV for 5 ms to allow inactivation to recovery to a steady-state (Scheme 1), followed by a return step to +60 mV (Fig 6).

**Scheme 1**.

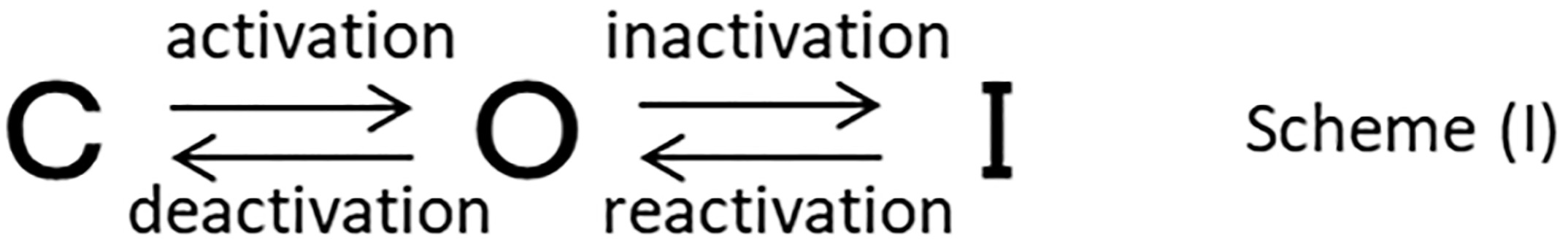


The peak of the tail currents plotted as a function of the preceding test pulse potentials were fitted with a Boltzmann function, namely the steady-state inactivation curve, was assessed as the reactivation kinetics. At more negative voltages from -170 mV to -200 mV, a proportion of the hERG channels also deactivate during the 5 ms test potential, leading the observed decrease in the peak *I*_*hERG*_ at negative potentials. Therefore, before fitting of the Boltzmann equation, this deactivation was corrected based on the pulse protocol in Fig 5. For analysis of the concentration dependency of gemcitabine on *I*_*hERG*_, we used six concentrations of gemcitabine (0.01, 0.05, 0.1, 1, 5, and 50 μM) to construct the half-maximal inhibitory concentration (IC_50_) curve, which was sigmoidal in shape plotted against the log [gemcitabine] fitted with the Hill equation.

### Gene expression analyses

The cDNA was synthesized from 1 μg of total RNA using the Transcriptor First Strand cDNA Synthesis Kit (Roche Molecular System Inc, Alameda, CA). Real-time PCR was performed on a LightCycler 96 system (Roche) using THUNDERBIRD Next SYBR qPCR (Toyobo, Osaka, Japan) as a detection reagent. Forward and reverse primer sequences respectively for the rat KCNH2 channel were designed from the sequence in the GenBank database as follows (accession numbers are indicated in parentheses): Kcnh2 (117018), forward 5’-TCAACCTGCGAGATACCAACATG-3’, reverse 5’-CTGGCTGCTCCGTGTCCTT-3’. Data were calculated by 2^-ΔΔCT^ and are presented as fold change induced in the transcript of each myocyte gene assayed by gemcitabine exposure. Gene expression was normalized to that of GAPDH and compared with the control condition (vehicle defined as 1.0).

### Western blotting

Western blotting was performed as described previously [23]. After 48 hours for gemcitabine and/or tunicamycin treatment, cardiomyocytes were lysed on ice 60 min in SDS-modified RIPA buffer containing protease inhibitor cocktails (Cytiva, Marlborough, MA, USA), and then centrifuged at 4 °C 15,000 rpm for 20 min. The resulting cell lysates (30 μg each) were boiled with SDS sample buffer and subjected to SDS-PAGE. The samples were transferred to a polyvinylidene difluoride membrane (Merk Millipore, Darmstadt, Germany), which was blocked for 1 h in Block Ace (DS Pharma, Osaka, Japan) at room temperature, then incubated overnight at 4 °C with primary antibodies against hERG (mouse monoclonal antibody, 1:100, sc-515611, Santa Cruz Biotechnology, Santa Cruz, CA, USA) and GAPDH (rabbit IgG-polyclonal antibody, 1:1000, 10494-1-AP, Proteintech, Rosemont, IL, USA). After washing with 1 × TBS containing 0.1% Tween 20, the membranes were incubated for 1 h at RT with appropriate secondary antibodies, followed by rewashing. Finally, the signals were detected using an ECL Western blotting analysis system (GE Healthcare, Piscataway, NJ, USA) in accordance with the manufacturer’s instructions. For detection of hERG and GAPDH, we performed two or three independent Western blots, and representative data are shown. The band intensities were quantified using Image J software (National Institute of Health, USA).

### Immunocytochemistry

The details of the experiments protocol were performed as described previously [15]. Primary antibodies against hERG (1:50, Santa Cruz) and α-actinin (1:200, Cell Signaling, Beverly, MA, USA) were applied following by incubation with the appropriate fluorescence-labeled secondary antibodies (Invitrogen) for 1 h at room temperature. After several washes, the samples were air-dried, mounted with a drop of ProLong Diamond Antifade Mountant with DAPI (Molecular Probes) and subjected to microscopy. Images were acquired using a 100× oil objective (Plan-Apochromat 100× [numerical aperture, 1.46] oil immersion objective for differential interference contrast [DIC]; Carl Zeiss). All sections were analyzed using a confocal laser microscopy system and software (LSM710; Carl Zeiss) that was built around an inverted microscope (Axio Observer Z1; Carl Zeiss, Germany). Images were saved in TIFF format and analyzed by ImageJ software (Wayne Rasband, National Institutes of Health, USA).

### Chemicals

All chemicals were purchased from Wako Pure Chemical Industries (Osaka, Japan). Gemcitabine was dissolved in dimethyl sulfoxide (DMSO) as stock solutions (50 mM), and then diluted to final concentrations in cell culture solutions. The final concentration of DMSO or acetic acid in the bathing solution was 0.01% or less.

### Statistical analysis

All data are presented as mean ± S.E. Statistical analysis was performed by one-way ANOVA with Tukey-Kramer test, Dunnett’s post hoc test when comparing every experimental group mean to the control group mean, or non-parametric Steel-Dwass test when data was not normal. To assess the differences in two groups, unpaired Student’s *t* test was performed. EC_50_ values were estimated by using non-linear least square curve-fitting programs in Sigma plot software ver.10 (SPSS, Chicago, IL, USA). Differences were considered significant when *p* values were less than 0.05 if nothing else is mentioned.

## Results

### Acute- and long-term effect t of gemcitabine on *I*_*hERG*_

We first examined the possible acute effect of gemcitabine on *I*_*hERG*_. hERG-HEK cells were superfused with normal Tyrode’s solution and the membrane potential was held at -80 mV followed by test pulses with a duration of 4 s, and then voltage-clamped at the potentials of -40 mV for 4 s (Fig 1, inset). Fig 1A illustrates typical acute effects of gemcitabine (50 μM) for 5 min on *I*_*hERG*_ family, demonstrating a negligible action of gemcitabine on *I*_*hERG*_. Effect of gemcitabine on the terminal *I*_*hERG*_ at the end of the test pulse of 4 s (indicated by hollow arrowheads) were plotted against the membrane potentials imposed (*I*_*hERG*.*term*_-*V* relationship), and summarized in Fig 1B. Effect of gemcitabine on the maximum tail *I*_*hERG*_ at the beginning of the pulse at -40 mV (indicated by filled arrowheads) following the test potentials were plotted against the membrane potentials imposed (*I*_*hERG*.*tail*_-*V* relationship), and summarized in Fig 1C. The amplitude of the maximum outward *I*_*hERG*.*term*_, the bell-shaped *I*_*hERG*.*term*_-*V* relationship or the maximum *I*_*hERG*.*tail*_ was unchanged by gemcitabine. According to these results, it is concluded that gemcitabine has substantially no actions on *I*_*hERG*_ as assessed at 5 min after application.

We then assessed the action of gemcitabine on *I*_*hERG*_ as applied in the culture medium for hERG-HEK cells for 24 h. Fig 1D indicates representative samples of *I*_*hERG*_ family recorded with or without actions of gemcitabine (50 μM) for 24 h, demonstrating a long-term inhibitory effect of gemcitabine on *I*_*hERG*_. Note that the solution was changed to gemcitabine-free bath solution prior to the patch-clamp study for 1 h. The *I*_*hERG*.*term*_-*V* relationship revealed a long-term reduction of *I*_*hERG*_ by gemcitabine for 24 h; terminal *I*_*hERG*_ was reduced by 53% (50 μM) as assessed by the maximum outward current at +10 mV or +20 mV (Fig 1E). The *I*_*hERG*.*tail*_-*V* relationship revealed a long-term reduction of *I*_*hERG*_ as well by gemcitabine for 24 h; the tail *I*_*hERG*_ was reduced by 52% (50 μM) as assessed by the maximum outward current at +60 mV (Fig 1F). Interestingly, the half-activation potential (*V*_*1/2*.*act*_) was significantly changed by gemcitabine treatment; *V*_*1/2*.*act*_ was 0.1 ± 0.8 mV in vehicle and -5.4 ± 1.4 mV by gemcitabine (50 μM) treatment, which suggests that gemcitabine not only suppresses *I*_*hERG*_ but also modifies the gating properties of the channel.

### Concentration-dependent suppression of *I*_*hERG*_ by gemcitabine

**Fig 2:**
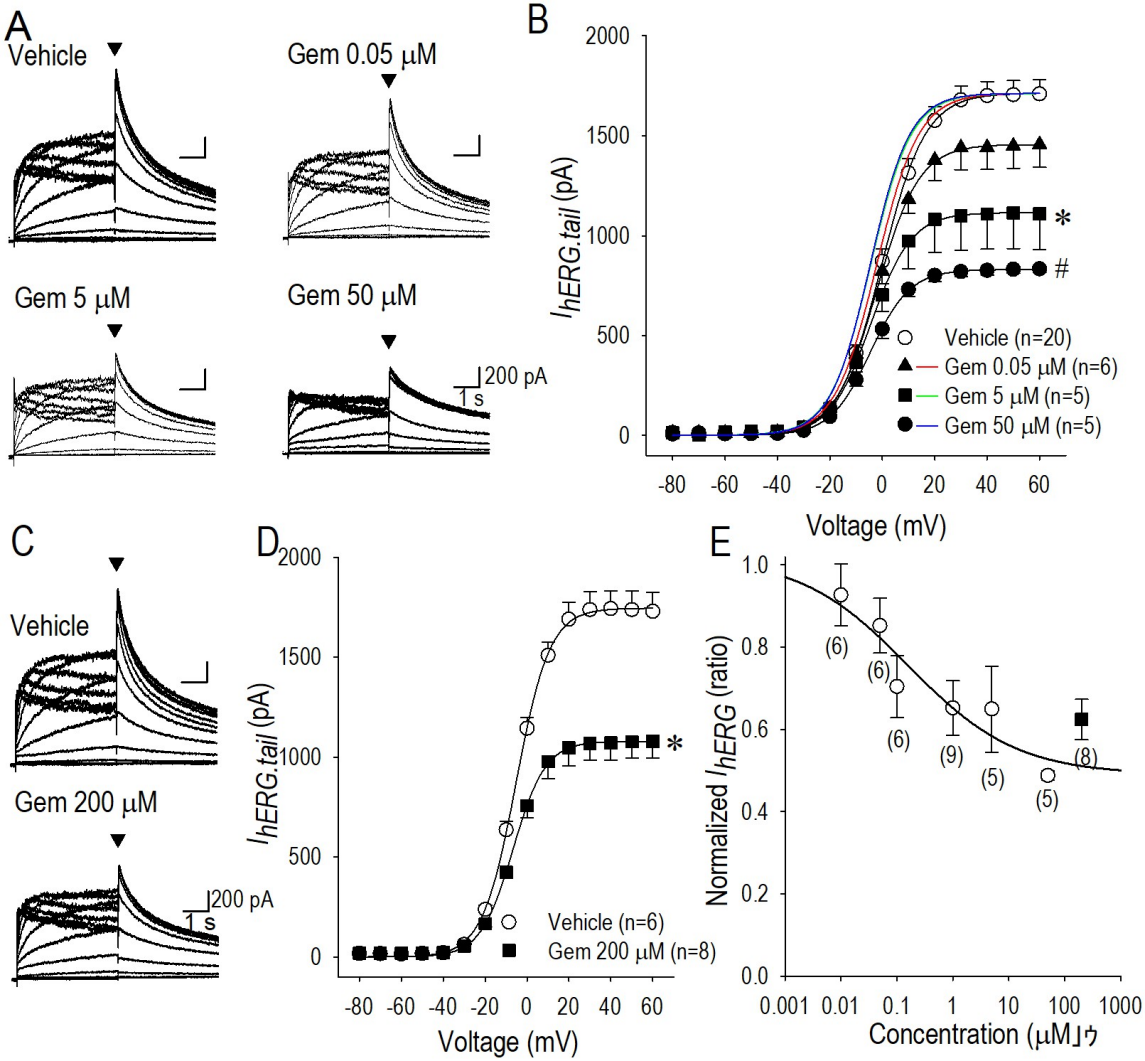
Dose-dependent inhibition of *I*_*hERG*_ by long-term application of gemcitabine. (A) Representative *I*_*hERG*_ family traces in vehicle, 0.05 μM, 5 μM and 50 μM gemcitabine (Gem) applied for 24 h. Pulse protocol was identical to that shown in Fig 1A and 1D. (B) The *I*_*hERG*.*tail*_-*V* relationship constructed by using group data in vehicle (〇) and long-term application of 0.05 μM (▲), 5 μM (■) and 50 μM gemcitabine (●) for 24 h. *I*_*hERG*.*tail*_ was assessed by the amplitude of the tail current of *I*_*hERG*_ indicated by the filled arrowheads in panel (A). Red line (Gem 0.05 μM), green line (Gem 5 μM) and blue line (Gem 50 μM) represent Boltzman-fitting curve of each gemcitabine data normalized to the *I*_*hERG*.*tail*_ in vehicle at +60 mV. (C) Representative *I*_*hERG*_ family traces in vehicle and 200 μM gemcitabine applied for 2 h. In this application protocol, hERG-HEK cells were incubated with 200 μM gemcitabine for 2 h, followed by incubation with gemcitabine-free culture medium for 22 h. (D) The *I*_*hERG*.*tail*_-*V* relationship constructed by group data in vehicle (〇) and 200 μM gemcitabine (■) applied by the application method described in panel (C). The maximum *I*_*hERG*.*tail*_ was reduced by 38% from 1728 ± 96 pA (vehicle) to 1079 ± 84 pA (gemcitabine). (E) Fractional changes in *I*_*hERG*_ measured at +60 mV in *I*_*hERG*.*tail*_ were plotted against the concentration of gemcitabine applied for 24 h (〇) and 2 h (■) based on the method mimicking clinical application protocol as described in panel (C). Numbers of cell are shown in parentheses. Values represent the mean ± SE. Statititical significance was determined by ANOVA with Dunnett test (Panel B) and unpaired Student’s t test (Panel D).

Dose-dependent suppression of *I*_hERG_ by gemcitabine was constructed by measuring percentage suppression of the maximum tail current of *I*_hERG_ with various concentration of gemcitabine (Fig 2). As indicated by representative current families of *I*_*hERG*_ (Fig 2A), the hERG-HEK cells display lower *I*_*hERG*_ amplitude when gemcitabine concentration was increased. The *I*_*hERG*.*tail*_-*V* relationship revealed a clear dose-dependency of gemcitabine actions on inhibition of *I*_*hERG*_ (Fig 2B). Importantly, V_1/2.act_ was also changed in a concentration-dependent manner; V_1/2.act_ was 0.1 ± 0.9 mV in vehicle, -2.6 ± 1.7 mV by 0.05 μM gemcitabine, -5.2 ± 1.8 mV by 5 μM gemcitabine and -5.4 ± 1.4 mV by 50 μM gemcitabine. Dose-response curve of *I*_*hERG*.*tail*_ suppression by gemcitabine indicates that the median effect of concentration (EC_50_) of gemcitabine to suppress *I*_*hERG*_ was 0.16 μM (Fig 2E).

According to the standard clinical methods for gemcitabine application protocol by which patient is being treated, 1,000–1,250 mg/m^2^ of gemcitabine is to be injected intravenously for 20–30 min once a week [24]. By this application protocol, the maximum serum concentration of gemcitabine reaches approximately 200 μM immediately after the injection [25], which follows the concentration declining period for nearly 2 h. Thus, we mimicked the application protocol of gemcitabine in this *in vitro* experiment by culturing hERG-HEK cells with 200 μM gemcitabine for 2 h, followed by incubation with gemcitabine-free medium for 22 h prior to the patch-clamp study. With this experimental protocol, *I*_*hERG*_ was suppressed nearly by 29% in the representative *I*_*hERG*_ family (Fig 2C), and by 38% in the group data (Fig 2D). The result of the normalized *I*_*hERG*_ with this application mimicking clinical protocol is compatibly represented in Fig 2E (filled square).

### Actions of gemcitabine on *I*_*hERG*_ activation

**Fig 3:**
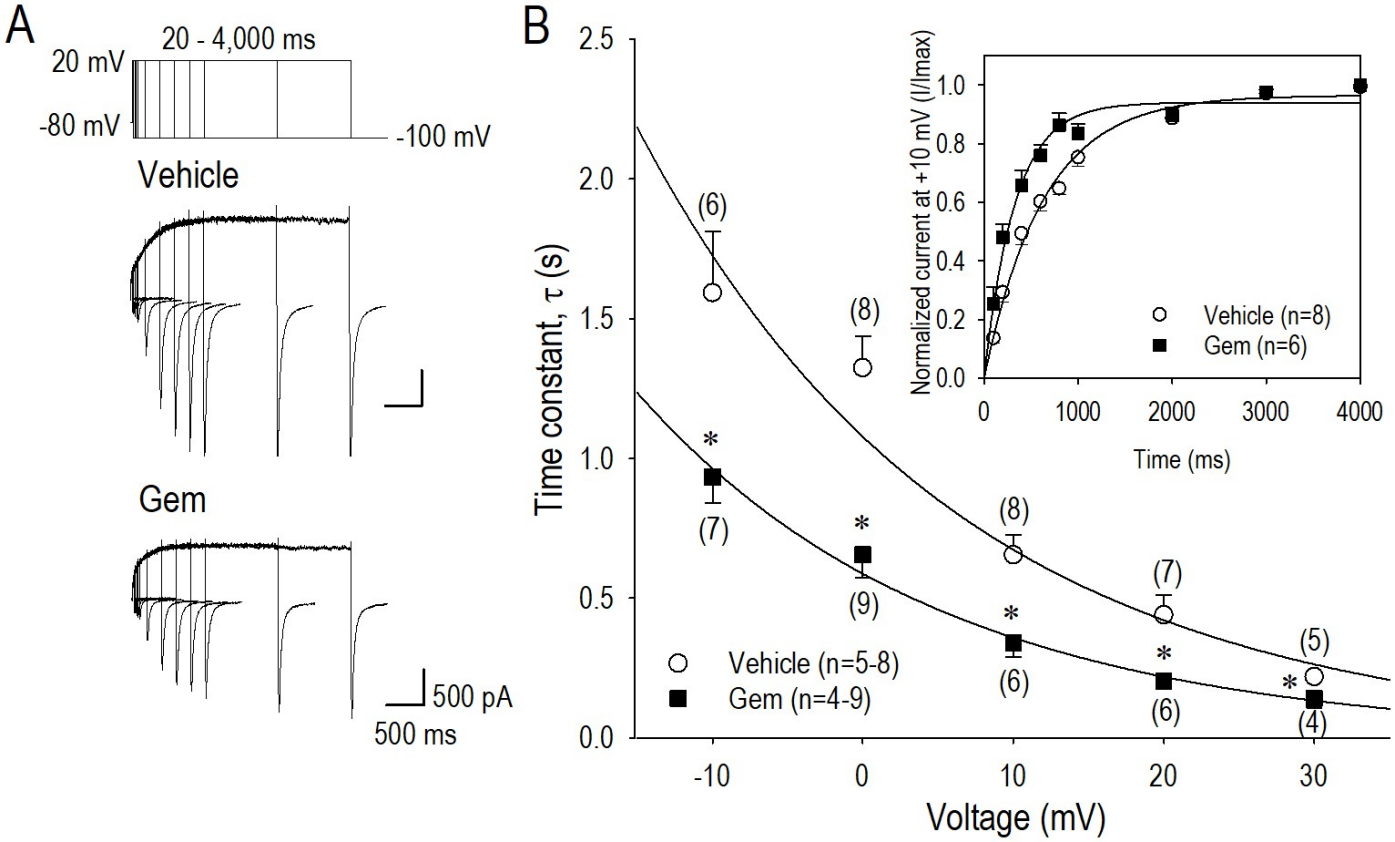
Actions of gemcitabine on *I*_*hERG*_ activation gating. (A) Representative *I*_*hERG*_ traces elicited by pulse protocol shown above, which displays time course of *I*_*hERG*_ activation measures by envelope of tail test in vehicle and in 0.05 μM gemcitabine (Gem) for 24 h. (B) Activation time constant (τ_act_)-V relationship in vehicle (〇) and gemcitabine (■), where τ_act_ at each test potential was obtained by a single exponential fitting curve in a plot of pulse duration (20–4,000 ms) versus *I*_*hERG*.*tail*_. An inset shows a fitting curve of the activating *I*_*hERG*_ at the potentials of +10 mV, yielding τ_act_ of 0.66 s for vehicle and of 0.34 s for gemcitabine. Data of τ_act_ were further fitted by a single exponential curve (see Methods Section), which yields *I*_*hERG*_ activation voltage constant of 21.3 mV for vehicle (〇) and 20.2 mV for gemcitabine (■). Numbers of cell are shown in parentheses. Values represent the mean ± SE. Unpaired Student’s *t* test was performed. *p<0.05 vs. Vehicle.

Long-term actions of gemcitabine on *I*_*hERG*_ activation were examined using the envelope of the tail test in hERG-HEK cells treated with vehicle or 0.05 μM gemcitabine for 24 h (Fig 3). Fig 3A indicates representative *I*_*hERG*_ in vehicle and in gemcitabine corresponding to the pulse protocol shown above. Then the normalized amplitude of the tail *I*_*hERG*_ was plotted against the pulse duration, yielding the time course of activation process at the test potentials (Fig 3B). In Fig 3B inset, the time course of *I*_*hERG*_ activation at the test potential of +10 mV was fitted to a single exponential function in vehicle and gemcitabine. Activation time constant (τ_act_) at the potentials of +10 was 0.66 ± 0.07 s in vehicle and 0.34 ± 0.05 s in gemcitabine. As demonstrated in τ_act_-*V* relationship in Fig 3B, activation of the hERG channel was shifted to the direction of hyperpolarization; the shift was 12.5 mV as assessed at the activation time constant (τ_act_) value of 1.0 s.

### Actions of gemcitabine on *I*_*hERG*_ inactivation

**Fig 4:**
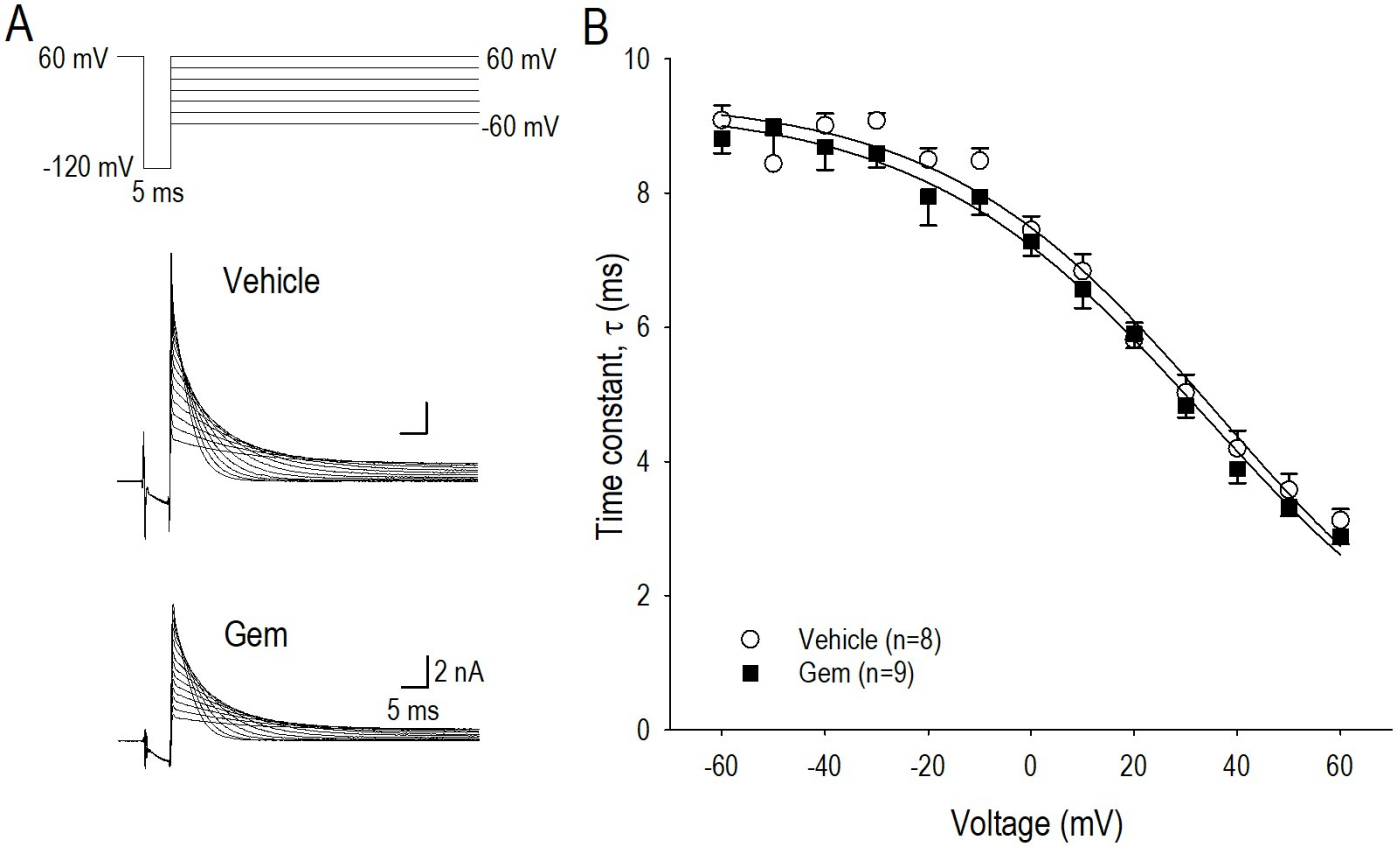
Actions of gemcitabine on *I*_*hERG*_ inactivation gating. (A) Representative *I*_*hERG*_ traces elicited by pulse protocol shown above, which displays voltage-dependent decay or inactivation of *I*_*hERG*.*tail*_ in vehicle and 0.05 μM gemcitabine (Gem) for 24 h. (B) Relationship between time constant of the decay phase of *I*_*hERG*.*tail*_ or τ_inact_ and the test pulse potential in vehicle (〇) and gemcitabine (■). Numbers of cell are shown in parentheses. Values represent the mean ± SE.

Long-term actions of gemcitabine on *I*_*hERG*_ inactivation were assessed by applying a conditioning pulse to +60 mV for 1s followed by a brief hyperpolarizing pulse (-120 mV for 5 ms) to allow the hERG channel to recover from inactivation (Fig 4). Depolarizing test pulses were applied to record inactivating *I*_*hERG*_. Fig 4A indicates representative *I*_*hERG*_ in vehicle and gemcitabine corresponding to the pulse protocol shown above, where the time course of fast inactivating *I*_*hERG*_ was fitted to a single exponential function, yielding a plot demonstrating voltage dependency of the fast time constant of *I*_*hERG*_ inactivation (τ_inact_) (Fig 4B). Gemcitabine substantially unaffected τ_inact_ at all examined potentials, suggesting that long-term modification of the hERG channel by gemcitabine could not regulate gating properties of the channel from the open state to the inactivation state.

### Actions of gemcitabine on *I*_*hERG*_ deactivation

**Fig 5:**
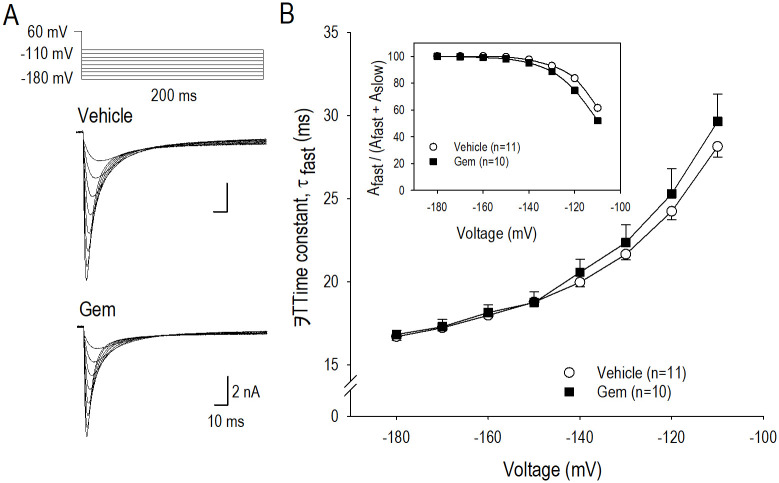
Actions of gemcitabine on *I*_*hERG*_ deactivation gating. (A) Representative *I*_*hERG*_ traces elicited by pulse protocol shown above, which displays deactivating phase of *I*_*hERG*_ in vehicle and 0.05 μM gemcitabine (Gem) for 24 h. (B) Relationship between time constant of the decay phase of *I*_*hERG*_ or τ_deact_ and the test pulse potential in vehicle (〇) and gemcitabine (■). An inset shows a ration of the fast component of the decay amplitude (A_fast_) to whole decay amplitude (A_fast_ + A_slow_). Although the decaying phase was fitted by a sum of two exponential components, τ for the fast component or the main component of the decay phase was assessed as τ_deact_ for the deactivating gating properties. Numbers of cell are shown in parentheses. Values represent the mean ± SE.

Deactivation process of the hERG channel was analyzed by applying long hyperpolarizing test pulses after a conditioning pulse to +60 mV (Fig 5A, inset) to assess the long-term actions of gemcitabine. Fig 5A demonstrates representative I_hERG_ in vehicle and gemcitabine corresponding to the pulse protocol shown above, where the decay phase of instantaneous *I*_*hERG*_ at the beginning of the test pulse could be fitted to a double exponential function. Although the fast phase could be ascribed to the mixture of both inactivation and the deactivation steps based on this pulse protocol, inactivating *I*_*hERG*_ with an extremely fast gating process may has little impact on the decay of *I*_*hERG*_ [9]. Because of this reason, the fast component of the time constant was compared as a parameter of channel deactivation (τ_deact_) between vehicle and gemcitabine treatment. Gemcitabine treatment of hERG-HEK cells did not affect τ_deact_ of *I*_*hERG*_ suggesting that gemcitabine has no action on deactivation of the hERG channel as a long-term effect. Namely, long-term modification of the hERG channel by gemcitabine could not regulate gating properties of the channel from the open state to the closed state. Analysis for the fast decay phase amplitude (A_fast_) also supports the conclusion (Fig 5B, inset).

### Actions of gemcitabine on *I*_*hERG*_ reactivation

**Fig 6:**
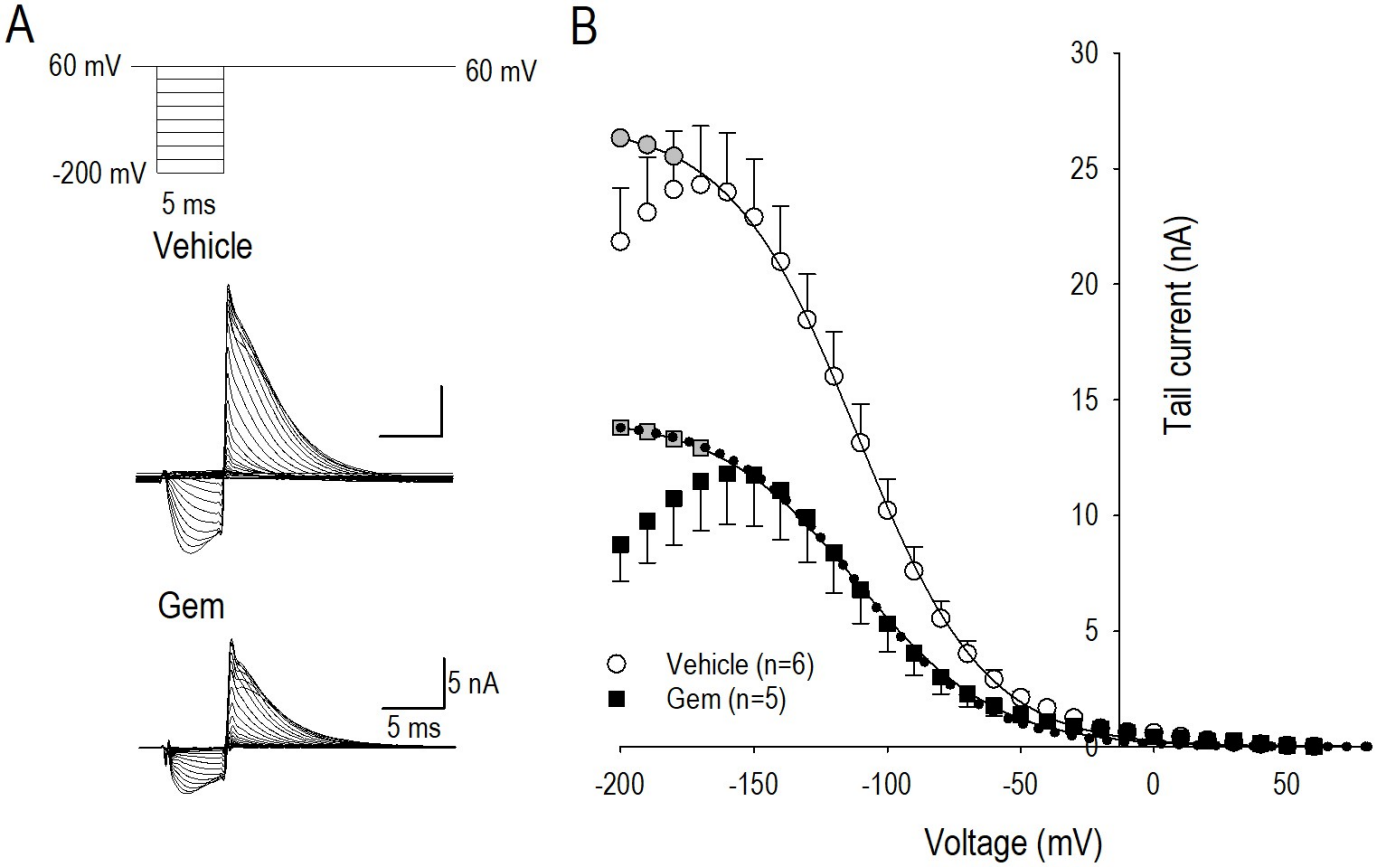
Actions of gemcitabine on *I*_*hERG*_ reactivation gating. (A) Representative *I*_*hERG*_ traces elicited by pulse protocol shown above, which displays reactivating *I*_*hERG*_ in response to the conditioning potentials (-200 mV—+60 mV) in vehicle and 0.05 μM gemcitabine (Gem) for 24 h. (B) Relationship between amplitude of reactivating *I*_*hERG*.*tail*_ and the conditioning pulse potentials in vehicle (〇) and gemcitabine (■). Gray filled circles and gray filled squares represent corrected data set after the replacement of *I*_*hERG*_ deactivating component. Each solid curve was a product of a fit by Boltzmann equation, and dotted line was drawn by Boltzmann fitting curve for vehicle data multiplied by 0.45 that representing the ratio (gemcitabine-*I*_*hERG*_ / vehicle-*I*_*hERG*_) at -200 mV. Numbers of cell are shown in parentheses. Values represent the mean ± SE.

Comparison of the process at the recovery from inactivation or reactivation of the hERG channel in vehicle and by gemcitabine treatment was performed by use of the standard three-stage voltage protocol. A 2-s pulse to +60 mV was first applied to attain steady-state inactivation, which was followed by a series of test pulses ranging from -200 mV to +60 mV for 5 ms (Fig 6A inset). This pulse duration is enough to allow recovery of the channel from inactivation, but too short to allow deactivation. Fig 6A demonstrates representative *I*_*hERG*_ in vehicle and gemcitabine treatment corresponding to the pulse protocol shown above, where the peak of the third pulse current was plotted against the potentials of the second pulses, yielding reactivation *I-V* curves (Fig 6B). At more negative voltages than -170 mV, a proportion of the channels also deactivate during the second pulse, leading to the observed decrease in the peak currents. Before fitting of the Boltzmann equation, this deactivating current was corrected based on the separate protocol such as that shown in Fig 5A to obtain the rates of deactivation at each voltage in the second pulse. With this analysis, voltage dependence of the recovery from steady-state-inactivation or reactivation of *I*_*hERG*_ in vehicle and gemcitabine was nearly identical (Fig 6B), which suggests that modification of the hERG channel by gemcitabine does not affect the reactivation process of the channel. Namely, long-term modification of the hERG channel by gemcitabine could not regulate gating properties of the channel from the inactivated state to the open state.

### Actions of gemcitabine under the inhibition of glycosylation

**Fig 7:**
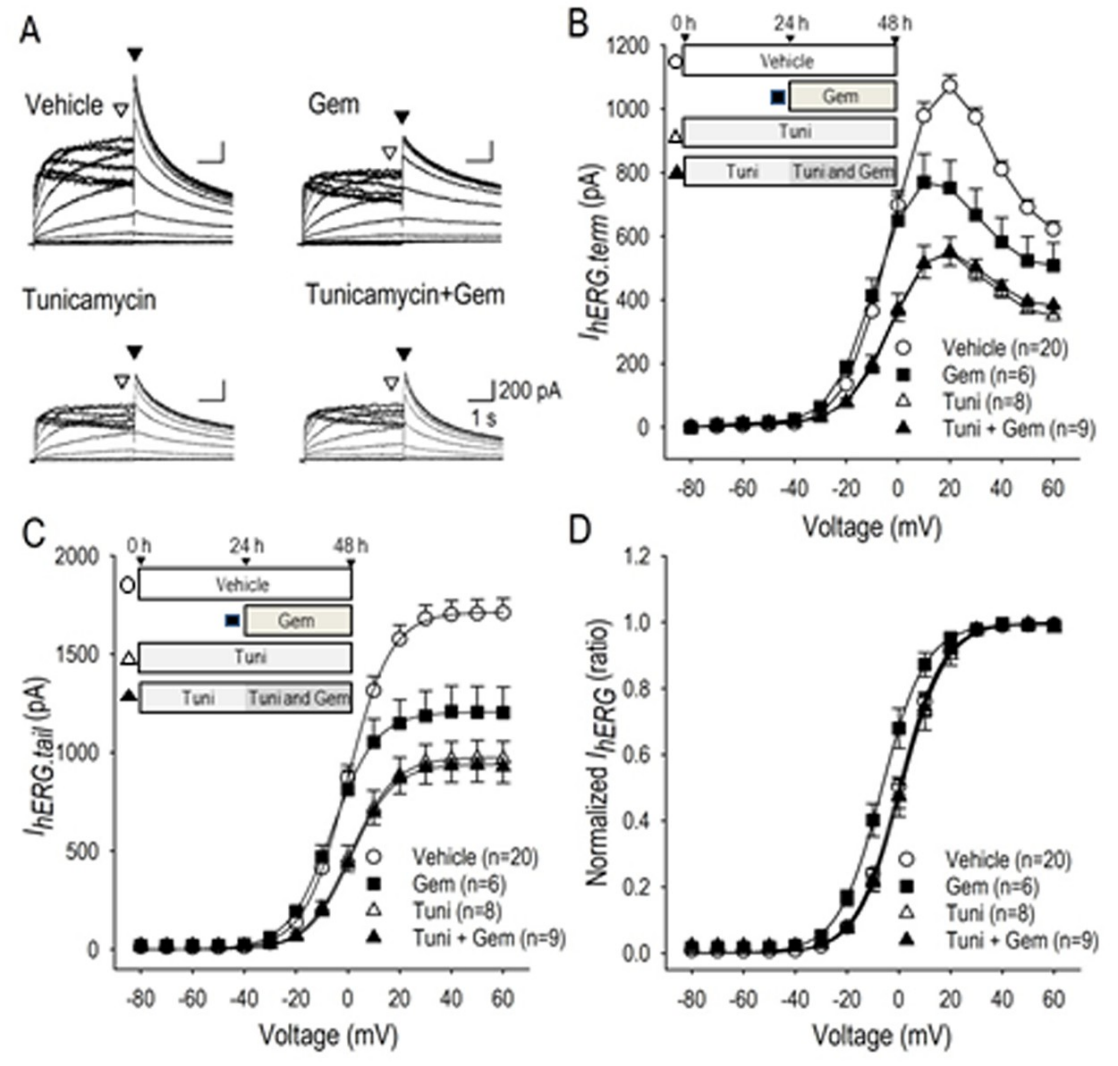
Actions of gemcitabine on *I*_*hERG*_ in combined with tunicamycin. (A) Representative *I*_*hERG*_ traces in vehicle (48 h), 0.1 μM gemcitabine (Gem) (24 h), 0.5 μg/ml tunicamycin (Tuni) (48 h), and 0.1 μM gemcitabine (24 h) with 0.5 μg/ml tunicamycin (48 h). Tunicamycin was applied to the culture medium 24 h prior to and during the application of gemcitabine for 24 h. (B) The relationship between *I*_*hERG*.*term*_ measured at the end of the test pulse (indicated by hollow arrowheads in panel A, ▽) and test pulse potential (V) constructed by using group data in vehicle (〇), 0.1 μM gemcitabine (■), 0.5 μg/ml tunicamycin (△), and gemcitabine with tunicamycin (▲). (C) The relationship between *I*_*hERG*.*tail*_ measured at the beginning of the third pulse at -40 mV (indicated by filled arrowheads in panel A, ▼) and test pulse potential (V) constructed by using group data are shown as *I*_*hERG*.*tail*_-*V* relationship in vehicle (〇), 0.1 μM gemcitabine (■), 0.5 μg/ml tunicamycin (△), and gemcitabine with tunicamycin (▲). (D) Normalized *I*_*hERG*.*tail*_–*V* relationship based on data in panel C for vehicle (〇), 0.1 μM gemcitabine (■), 0.5 μg/ml tunicamycin (△), and gemcitabine with tunicamycin (▲). V_1/2_ was 0.1 ± 0.1 mV for vehicle, 6.3 ± 2.0 mV for gemcitabine, 1.2 ± 2.2 mV for tunicamycin and 0.8 ± 1.2 mV for tunicamycin with gemcitabine. Cell culture protocol is shown in inset. Numbers of cell are shown in parentheses. Values represent the mean ± SE. Statititical significance was determined by ANOVA with Tukey-Kramer test.

To explore the possible mechanism of gemcitabine on the hERG channel regulation, we examined the long-term actions of gemcitabine under the influence of asparagine (*N*)-linked glycosylation inhibition, because altered N-glycosylation levels of circulating glycoproteins were reported in cancer patients with gemcitabine treatment [26]. Also because N-glycosylation reportedly stabilizes hERG channel proteins on the plasma membrane [27], we recorded *I*_*hERG*_ in hERG-HEK cells treated with gemcitabine in the presence or absence of the inhibitory signals for N-linked glycosylation. Tunicamycin is widely used as a research tool to block N-linked glycosylation by blocking the transfer of N-acetylglucosamine 1-phosphate to dolichol monophosphate. Fig 7A demonstrates representative *I*_*hERG*_ in vehicle, in gemcitabine alone (24 h), in tunicamycin alone (48 h), and in gemcitabine (24 h) combined with tunicamycin treatment (48 h) in the culture medium; tunicamycin was applied to the culture medium 24 h prior to and during the gemcitabine application. Of note, gemcitabine was without effect on *I*_*hERG*_ when *I*_*hERG*_ was highly suppressed by the co-presence of tunicamycin. When terminal *I*_*hERG*_ at the end of each test pulse were plotted against the voltage applied, a bell-shaped relationship was maintained in treatment of gemcitabine, tunicamycin, and gemcitabine combined with tunicamycin, although the potential for the maximum outward current was shifted by the treatment of gemcitabine (Fig 7B), which is consistent with the result in Fig 1E. It is also notable that the differences of the shape of terminal *I*_*hERG*_*-V* relationship (Fig 7B) and the tail *I*_*hERG*_ *-V* relationship (Fig 7C) between tunicamycin and gemcitabine combined with tunicamycin was negligibly small, which suggests that actions of gemcitabine on the hERG channel and actions for the N-glycosylation disruption overlaps significantly. We also noted that gemcitabine treatment significantly shifted the normalized tail *I*_*hERG*_ toward the hyperpolarized direction by 6.4 mV, while gemcitabine was unable to shift the tail *I*_*hERG*_ when hERG-HEK cells were simultaneously treated with tunicamycin. These results suggest that action site of gemcitabine and tunicamycin on the hERG channel may not be identical.

**Fig 8:**
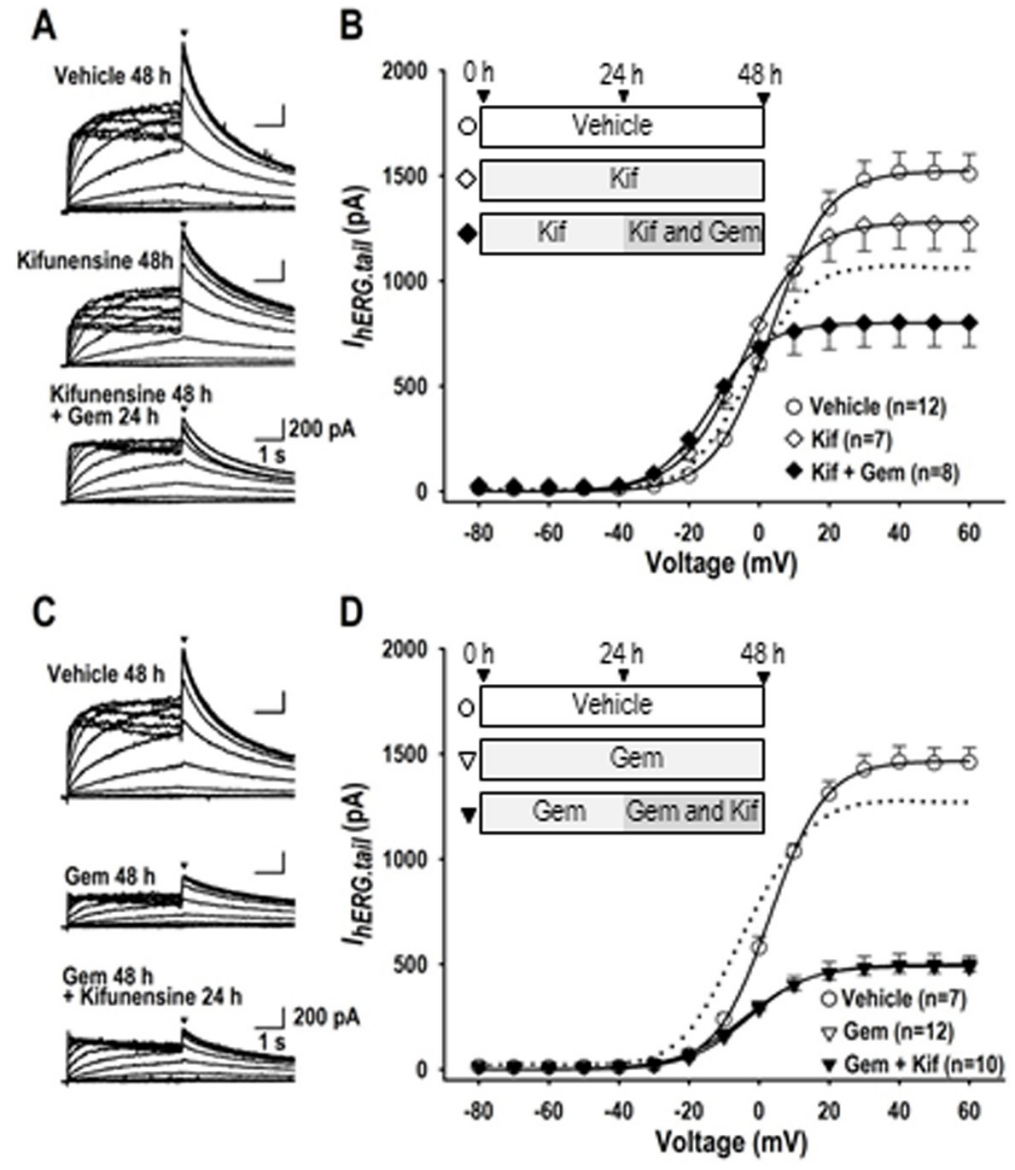
Actions of gemcitabine on *I*_*hERG*_ in combined with kifunensine. (A) Representative *I*_*hERG*_ traces in vehicle (48 h), 100 μM kifunensine (Kif) (48 h), and 100 μM kifunensine (48 h) with 0.1 μM gemcitabine (24 h). Kifunensine was applied to the culture medium 24 h prior to and during the application of gemcitabine for 24 h. (B) The relationship between *I*_*hERG*.*tail*_ measured at the beginning of the third pulse at -40 mV (indicated by filled arrowheads in panel A, ▼) and test pulse potential (V) constructed by using group data are shown as *I*_*hERG*.*tail*_-*V* relationship in vehicle (〇), 100 μM kifunensine (◇), 100 μM kifunensine with 0.1 μM gemcitabine (◆), and 0.1 μM gemcitabine (■). (C) Representative *I*_*hERG*_ traces in vehicle (48 h), 0.1 μM gemcitabine (48 h), and 0.1 μM gemcitabine (48 h) with 100 μM kifunensine (24 h). (D) The relationship between *I*_*hERG*.*tail*_ measured at the beginning of the third pulse at -40 mV (indicated by filled arrowheads in panel A, ▼) and test pulse potential (V) constructed by using group data are shown as *I*_*hERG*.*tail*_-*V* relationship in vehicle (〇), 0.1 μM gemcitabine (▽), 0.1 μM gemcitabine with 100 μM kifunensine (▼), and 100 μM kifunensine (◇). Gemcitabine was applied to the culture medium 24 h prior to and during the application of kifunensine for 24 h. Cell culture protocol is shown in inset. Numbers of cell are shown in parentheses. Values represent the mean ± SE. Statistical significance was determined by ANOVA with Tukey-Kramer test.

To further characterize the effect of gemcitabine on the hERG channel glycosylation disruption, a specific class I mannosidase inhibitor kifunensine [28] was applied in combined with gemcitabine. Fig 8A demonstrates representative *I*_*hERG*_ in vehicle (48 h), in kifunensine alone (48 h), and in kifunensine (48 h) combined with gemcitabine treatment (24 h). Importantly, gemcitabine further reduced *I*_*hERG*_ when combined with kifunensine. The tail *I*_*hERG*_*-V* relationship revealed that effect of gemcitabine on *I*_*hERG*_ was additive to the effect of kifunensine because the inhibition ratio of *I*_*hERG*_ at the voltage of +60 mV by gemcitabine was nearly identical between the conditions with (-35.8%) or without the presence of kifunensine (-30.5%). On the contrary, kifunensine was unable to reduce *I*_*hERG*_ when hERG-HEK cells were pretreated with gemcitabine for 24 h. These results suggest that *I*_*hERG*_ reduction caused by gemcitabine revealing a down-regulation of *I*_*hERG*_ through the post-translational glycosylation disruption possibly at the early phase of hERG channel glycosylation prior to mannosidase I-dependent process in the endoplasmic reticulum.

### Actions of gemcitabine on cardiomyocytes

**Fig 9:**
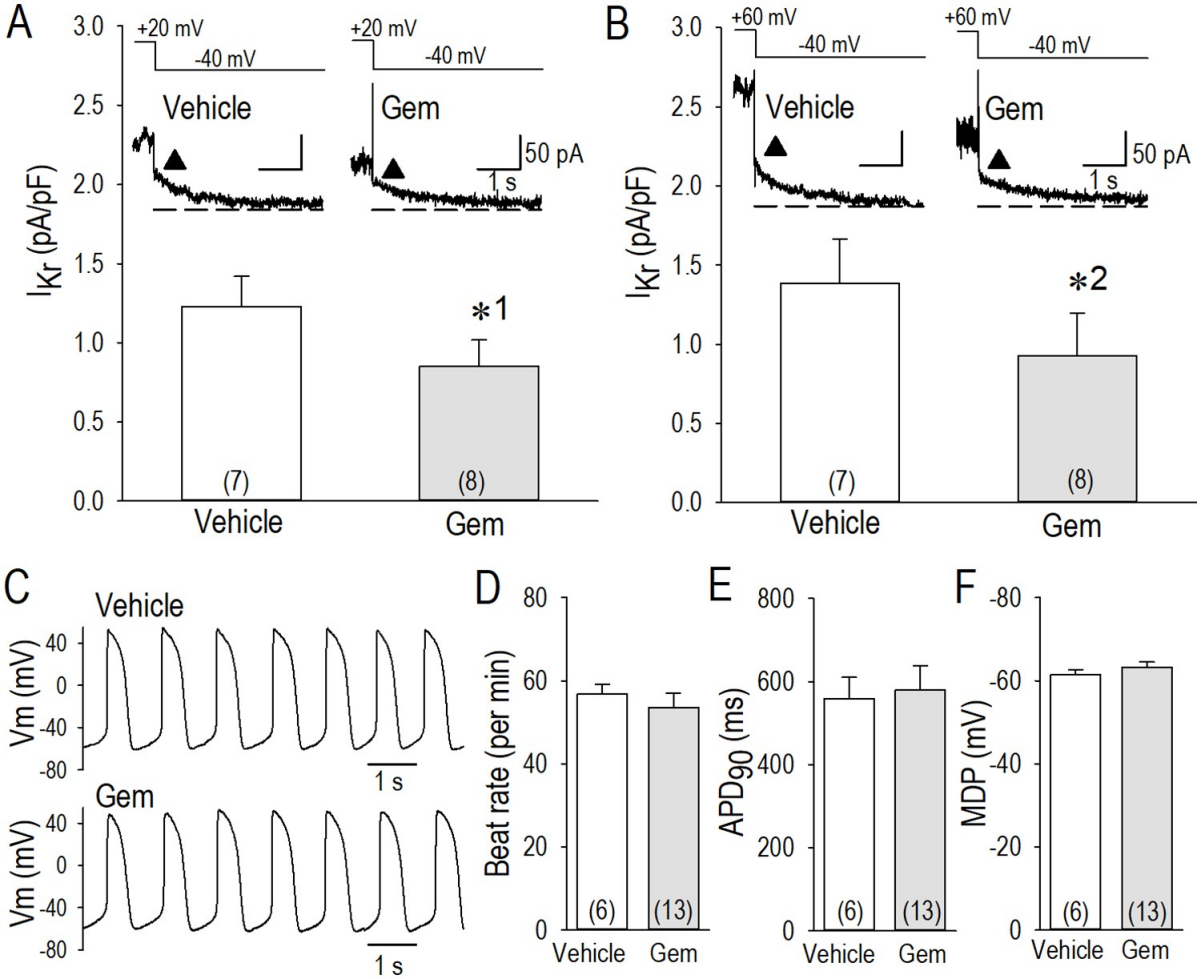
Long-term effects of gemcitabine on rapidly activating delayed rectifier K^+^ channel current (I_Kr_) and action potential configuration in neonatal rat cardiomyocytes. (A, B) Representative I_Kr_ tail current traces and group data of the control (vehicle) and long-term (24 h) application of gemcitabine (0.1 μM), corresponding to the pulse protocol shown above. Isolation I_Kr_ was obtained by subtraction of the currents recorded in the presence of E-4031 (10 μM) from the control currents. The maximum outward tail currents of I_Kr_ (indicated by filled triangles) were assessed as group data. (C) Representative action potentials in vehicle (24 h) and gemcitabine (24 h) of 0.1 μM. (D, E, F) Group data for cardiomyocyte spontaneous beating rate (D), action potential duration at 90% repolarization (APD_90_) (E), and the maximum diastolic potentials (MDP) (F). The Student’s t test was used to compare the means between two groups. Numbers of cell are shown in parentheses. Values represent the mean ± SE. Unpaired Student’s *t* test was performed. *p1 = 0.154, and *p2 = 0.260 vs. Vehicle.

Electrophysiological long-term actions of gemcitabine on neonatal cardiomyocytes were examined by voltage-clamp and current-clamp methods. Gemcitabine treatment for 24 h down-regulated E-4031-sensitive rapidly activating delayed rectifier K^+^ channel current (I_Kr_) in neonatal cardiomyocytes in a similar manner in hERG-HEK cells; gemcitabine appreciably reduced the tail current of I_Kr_ approximately by 30% when assessed by the pulse protocols at +20 mV and +60 mV (Fig 9A and 9B). Also note that the current density of I_Kr_ was as small as ~1 pA/pF in neonatal cardiomyocytes, which made it nearly unfeasible to assess the gating properties of I_Kr_ in detail. Meanwhile, the action potential configuration was changed only a little by gemcitabine treatment (Fig 9C–9F), because current density of I_Kr_ was very small in neonatal cardiomyocyte.

**Fig 10:**
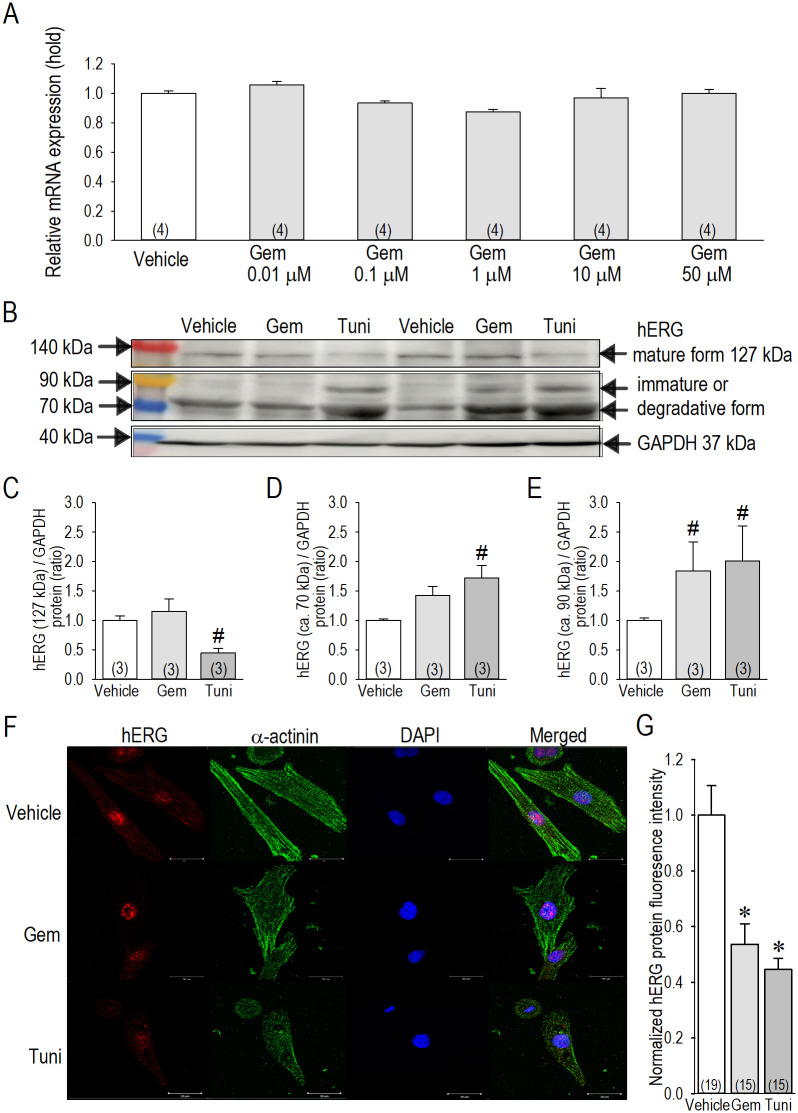
Molecular biological and immunocytochemical assay of gemcitabine actions on the hERG channel. (A) Changes of hERG mRNA expressions in vehicle and gemcitabine (0.01–50 μM) applied for 48 h were examined by qRNA. (B) Effects of 0.1 μM gemcitabine (48 h) on hERG protein expression evaluated by Western blot analysis. hERG protein levels of mature form of hERG protein (indicated by an arrow near 127 kDa) and immature/degradative form of hERG proteins (indicated by arrows near 90 kDa and 70 kDa). See the S1 File for the complete gel/blot images. (C, D, E) Normalization of Western blot data for hERG at 127 kDa (C), hERG at 70 kDa (D), and hERG at 90 kDa (E). Non-parametric Steel-Dwass test was applied. *P<0.1. (F) Expression and distribution of hERG as assessed by immunocytochemistry procedure. Cardiomyocytes were exposed to vehicle, gemcitabine (0.1 μM) or tunicamycin (0.5 μg/ml) for 24 h. hERG for stained in red, α-actinin in green, and DAPI staining to visualize nuclei in blue. Scale bar = 20 μm. (G) Quantitative results are shown. The fluorescence intensity of hERG protein with vehicle was set to 1.0. The result for each condition was obtained from different image slides indicated by parentheses. Statistical significance was determined by non-parametric Steel-Dwass test in Panels C, D, and E, whereas ANOVA with Tukey-Kramer test in Panel G. #*p* < 0.1 versus vehicle. *p < 0.01 versus vehicle. Values represent the mean ± SE.

In order to further support our conclusion that gemcitabine down-regulates hERG-I_Kr_ via inhibition of N-glycosylation pathway at N-acetylglucosamine (GlcNAc) phosphotransferase and/or the step(s) between the tunicamycin-sensitive and kifunensine-sensitive site, we additionally examined actions of gemcitabine on hERG mRNA and proteins in neonatal rat cardiomyocytes. It is of importance that gemcitabine substantially exerted no effect on hERG mRNA when applied for 48 h in cardiomyocytes (Fig 10A), suggesting that gemcitabine has no effect on the transcription of hERG. We then performed Western blot analyses to determine the impact of gemcitabine and tunicamycin on hERG protein expression in cardiomyocytes (Fig 10B). In the whole cell lysate of cardiomyocytes treated by tunicamycin, mature form of hERG (127 kDa band), which possibly carries full N-lined sugars, was significantly reduced in comparison with cardiomyocytes treated by vehicle (Fig 10C), whereas the possible immature forms of hERG proteins, indicated by bands near 70 kDa and 90 kDa, were increased by tunicamycin (Fig 10D and 10E). Interestingly, gemcitabine treatment upregulated possible immature forms of hERG protein indicated by bands at 70 kDa and 90 kDa, whereas it unaffected the mature form of hERG protein (127 kDa). Furthermore, immunocytochemistry staining revealed that gemcitabine and tunicamycin suppressed hERG protein expression nearly at the cell surface to a similar extent (Fig 10F and 10G), indicating that gemcitabine down-regulates hERG-I_Kr_ through post-translational pathway, likely to be involved in N-glycosylation step(s).

## Discussion

In the present study, we found that an anti-cancer drug gemcitabine suppressed *I*_*hERG*_ in heterologous hERG-HEK cells, as well as I_Kr_ in neonatal cardiomyocytes, as a long-term effect (24 h). The EC_50_ of gemcitabine to suppress *I*_*hERG*_ was 0.16 μM, which is within the serum concentration of gemcitabine after intravenous bolus injection based on the standard clinical application protocol [25]. As a long-term effect, gemcitabine modified the voltage dependency of the hERG channel activation, while inactivation, deactivation and reactivation gating properties were unaffected by gemcitabine. Gemcitabine treatment-related arrhythmias associated with QT prolongation or risk of torsade de pointes are possibly caused, at least in part, by the N-glycosylation disruption of the hERG channel.

### Gemcitabine-mediated arrhythmias

*I*_*Kr*_ or *I*_*hERG*_ plays a critical role in shaping action potentials in cardiomyocytes, especially the plateau phase and stabilizing action potential durations (APD). Reduction in *I*_*hERG*_ prolongs APD and effective refractory period (ERP), which may result in the destabilization and early termination of reentrant-based arrhythmias [22, 29]. However, cardiac arrhythmias were often reported in 10% of patients or more treated with gemcitabine [4]. Gemcitabine is actually known to cause atrial fibrillation [3, 4]. Indeed, high atrial fibrillation rate caused by gemcitabine was recorded, which may be associated with even the first dose [4]. These observations leave the focus of our attention on the question of how, if gemcitabine prolongs APD, it can also be involved in atrial mechanical function. To put the same question differently: If gemcitabine prolongs atrial contraction time, then how does the mechanism ensure that atrial fibrillation occurs in the case of gemcitabine application? The answer will depend on how atrial contraction regulates atrial/pulmonary vein pressure in human subject. According to human echocardiographic studies, in healthy individuals, left atrial contraction produces forward flow across the mitral valve, as well as a reverse flow in the pulmonary veins [30, 31]. Left ventricular cavity pressure and compliance during atrial contraction affect forward and reverse atrial flow [31, 32]. It is accordingly postulated that the reverse flow in the pulmonary veins will become more prominent when left atrial pressure is increased during atrial contraction. Because left atrial cardiomyocytes have large *I*_*Kr*_ density [33], suppression of I_Kr_ may participate in the prolongation of contraction time in the atrial tissue, which may cause the increase of atrial pressure and reverse flow in the pulmonary veins. Taken together, a transient suppression of *I*_*hERG*_ by gemcitabine could be a base for the mechanism of atrial fibrillation.

Although the incidence is not high, association of gemcitabine with QT interval prolongations were reported in several studies [6, 7, 34]. The timing of the QT interval at the organism level is related to the APD of ventricular cardiomyocytes acting at the cellular level [35]. Standing on these theoretical viewpoints, we speculate that gemcitabine decreases *I*_*hERG*_ in a long-term application to prolong APD, which could lead to QT prolongation and the risk of torsades de pointes-type arrhythmias. Therefore, caution is highly warranted to avoid conditions that can further lead to QT prolongation such as in hypokalemia, hypothermia, bradycardia, and class III antiarrhythmic drugs etc. in combined with gemcitabine treatment.

### Gemcitabine as a glycosylation modulator

Glycosylation of the hERG channels is known to N-linked at position N598 [36]. According to literature, glycosylation of the hERG channel is believed to increase hERG current and stabilize the channel protein on the plasma membrane by decreasing proteolytic susceptibility [37]. In this study, we inhibited N-linked glycosylation of the hERG channel in hERG-HEK cells with tunicamycin (10 mg/ml for 24 h) or kifunensine (100 μM for 24–48 h) to confirm the action of glycosylation on the hERG channel in combined with gemcitabine to explore the mechanism of the action. Because gemcitabine could not affect *I*_*hERG*_ when *I*_*hERG*_ was highly suppressed by tunicamycin (Fig 7C), it is plausible to conclude that gemcitabine acts to suppress a pathways of the channel glycosylation to reduce *I*_*hERG*_. More specifically, it is suggested that gemcitabine may act on the channel glycosylation step at the tunicamycin-sensitive GlcNAc phosphotransferase and/or the site between the tunicamycin-sensitive and kifunensine-sensitive process, because a mannosidase I inhibitor kifunensine was without effect on I_hERG_ when hERG-HEK cells were pretreated with gemcitabine for 24 h (Fig 8D). Since tunicamycin disrupts the formation of N-glycosidic linkages by inhibiting the first step in glycoprotein synthesis, and kifunensine blocks endoplasmic reticulum mannosidase I, which halts processing of glycoproteins and the transport to the Golgi, we assume that gemcitabine acts to disrupt the hERG channel glycosylation in the endoplasmic reticulum. Of note, gemcitabine up-regulated immature protein levels of hERG, and down-regulated cell surface expression of hERG. Because mature form of hERG was unchanged in Western blot analyses, and cell surface expression of hERG protein was significantly reduced as assessed by fluorescence intensity by gemcitabine treatment, it is suggested that gemcitabine may also affect the trafficking process of the hERG protein toward the plasma membrane. Taken all together with the result on hERG mRNA, it is likely to conclude that electrophysiological toxicity of gemcitabine on cardiomyocytes can be caused by post-translational disruption of the hERG channel.

We note the fact that disruption in glycan biosynthesis in some tumor cells could profoundly impact multiple molecular events and signaling pathways associated with anticancer drug resistance [38]. Actually, activation of endoplasmic reticulum stress and inhibition of N-glycosylation by tunicamycin enhances susceptibility of some cancer cells to anticancer drugs [39], which implies that some anticancer drugs may act to cancer cells somehow functionally related to protein glycosylation disruption. However, actions of gemcitabine may not be identical to that of tunicamycin on the hERG channel protein based on the fact that gemcitabine shifted the voltage dependency of *I*_*hERG*_ activation toward the hyperpolarized direction whereas tunicamycin was without effect on it (Fig 7D). Since gemcitabine modified the activation gating properties of the hERG channel (Fig 3), unknown long-term actions of gemcitabine on the hERG channel has been postulated accordingly.

### Limitation of the study

There are several limitations in the present study. Although we have demonstrated that gemcitabine has a potential to suppress *I*_*hERG*_ as a long term effect in heterologous expression system, we could not confirm the result on *I*_*Kr*_ in cardiomyocytes in detail. It is important to keep in mind that transcriptional, post-transcriptional and post-translation modulation pathways for hERG protein are quite different between heterologous hERG-HEK cells and cardiomyocytes. Also, the structure of glycosylation and its functional implications can vary significantly between different cell types, and between cultured cells and cardiac cells in vivo. Because an incubation technique for the cultivation of adult cardiomyocytes for the duration of 24 h or longer has not been established, we usually apply electrophysiological studies by use of neonatal cardiomyocyte for these purposes. However, neonatal cardiomyocytes only express very small density of *I*_*Kr*_ (Fig 9A and 9B), which is not suitable for the evaluation of the long-term effect of gemcitabine on the hERG channel [15]. Actually, gemcitabine was without effect on action potential configuration in neonatal cardiomyocytes (Fig 9C–9E), probably because I_Kr_ density was as small as ~1 pA/pF. As such, cardiac cells generated from iPS cells may be suitable for this analysis. Albeit we have obtained EC_50_ values of gemcitabine to suppress *I*_*hERG*_, a simple comparison of drug concentrations between in vitro study and human serum sample may not be appropriate. Because gemcitabine is a lipophilic drug, assessment of drug efficacy on *I*_*hERG*_ by an *in vitro* experiment without serum proteins and/or serum lipids may not represent the effect of gemcitabine in *in vivo* application. Furthermore, a caution would be needed to extrapolate these single cell experiments to human therapeutics. Regarding several glycosylation steps in the endoplasmic reticulum and in the Golgi apparatus, the first step of *N*-glycosylation in the assemblage of dolichol-linked precursor oligosaccharide at the cytoplasmic side of the endoplasmic reticulum could be blockaded by tunicamycin [40, 41]. As gemcitabine but not tunicamycin modified the activation gating of the hERG channel, and because a specific class I mannosidase inhibitor kifunensine was unable to reduce *I*_*hERG*_ when hERG-HEK cells were pretreated with gemcitabine, it is speculated that gemcitabine regulates some glycosylation step(s) between the GlcNAc phosphotransferase site andα-1, 2-mannosidase I site in the endoplasmic reticulum, although we were unable to identify the target molecule(s) of gemcitabine in the whole process of hERG protein glycosylation. Because this study is solely dependent upon electrophysiological evaluation of *I*_*hERG*_, molecular biological analyses for N-glycosylation are obviously needed to address these questions.

## Conclusions

Our study revealed that the long-term application of gemcitabine reduces *I*_*hERG*_ possibly through the inhibition of N-linked glycosylation of the channel, which may explain the hitherto unknown pharmacological mechanism of gemcitabine to cause QT prolongation in ECG. Furthermore, excessive reduction of *I*_*hERG*_ by gemcitabine in cardiomyocytes may account for supraventricular arrhythmogenicity of this drug as documented in clinical reports.

## Supporting information

S1 File (PDF)
