# Supplementary material for: Electrophysiological evaluation of an anticancer drug gemcitabine on cardiotoxicity revealing down-regulation and modification of the activation gating properties in the human rapid delayed rectifier potassium channel
Source: PLoS One. 2023 Feb 2;18(2):e0280656. doi: 10.1371/journal.pone.0280656 (PMC9894456; doi:10.1371/journal.pone.0280656)

Electrophysiological evaluation of an anticancer drug gemcitabine on cardiotoxicity revealing down-regulation and modification of the activation gating properties in the human rapid delayed rectifier potassium channel

Mengyan Wei [1,2†], Pu Wang [1,2†], Xiufang Zhu [1,2†], Masaki Morishima [3], Yangong Liu [1,2], Mingqi Zheng [1], Gang Liu [1], Hiroki Osanai [2], Kenshi Yoshimura [2] Shinichiro Kume [2] Tatsuki Kurokawa [2] and Katsushige Ono [2*]

[1] Department of Cardiology, The First Hospital of Hebei Medical University, 89 Donggang Road, Shijiazhuang, Hebei Province 050031, People's Republic of China;

[2] Department of Pathophysiology, Oita University School of Medicine, Yufu, Oita 879-5593, Japan.

[3] Department of Department of Food Science and Nutrition, Faculty of Agriculture, Kindai University, Nara, 631-8505, Japan.

Supporting Supplemental Figure
Effects of 0.1 μM gemcitabine (24 h) on hERG protein expression evaluated by Western blot analysis. hERG protein levels of mature form of hERG protein (indicated by red marker of 140 kDa) and immature/degradative form of hERG proteins (indicated by yellow marker of 90 kDa and blue marker below yellow marker of 70 kDa). Experimental protocols are described in the Methods Section in the manuscript. Upper panel and lower panels are identical with different exposure time. From left lane to right lane; vehicle, gemcitabine, tunicamycin, vehicle, gemcitabine, tunicamycin, vehicle, gemcitabine, blank, tunicamycin.

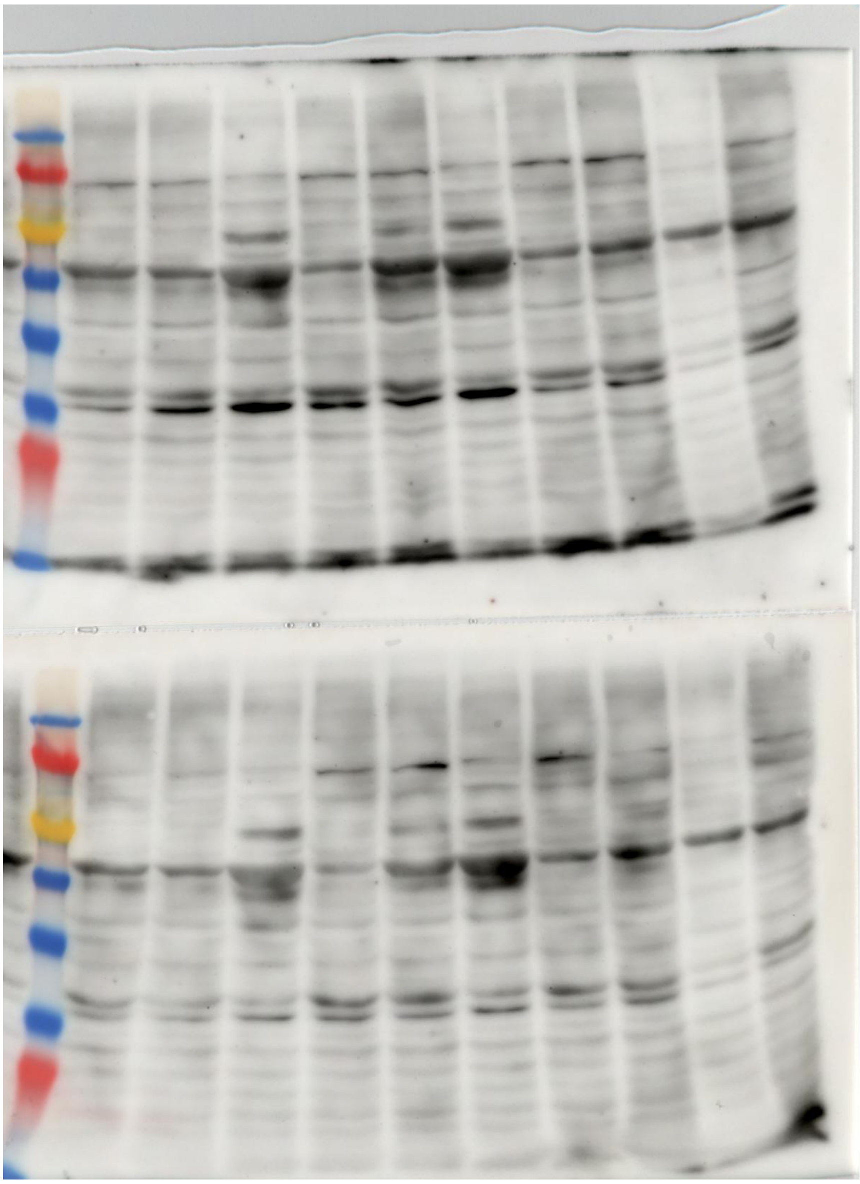

Supplement: S1 File — (PDF) [file pone.0280656.s001.pdf]
